# One-stage posterior surgery combined with anti-Brucella therapy in the management of lumbosacral brucellosis spondylitis: a retrospective study

**DOI:** 10.1186/s12893-022-01847-x

**Published:** 2022-11-18

**Authors:** Haopeng Luan, Kai Liu, Xiaonan Deng, Weibin Sheng, Mardan Mamat, Hailong Guo, Huaqiang Li, Qiang Deng

**Affiliations:** 1 Department of Spine Surgery, The First Affiliated Hospital of Xinjiang Medical University, Ürümqi, 830054 Xinjiang China; 2 Department of Trauma and Microreconstructive Surgery, The First Affiliated Hospital of Xinjiang Medical University, Ürümqi, 830054 Xinjiang China; 3 Department of Orthopaedic Surgery, Chengdu Xinhua Hospital Affiliated to North Sichuan Medical College, Chengdu, 610055 Sichuan China

**Keywords:** Brucellosis, Infection, Lumbosacral, Spine, Surgery

## Abstract

**Background:**

This study aimed to assess the clinical efficacy of one-stage posterior surgery combined with anti-Brucella therapy in the treatment of lumbosacral brucellosis spondylitis (LBS).

**Methods:**

From June 2010 to June 2020, the clinical and radiographic data of patients with LBS treated by one-stage posterior surgery combined with anti-Brucella therapy were retrospectively analyzed. The visual analogue scale (VAS), Japanese Orthopaedic Association (JOA) and Oswestry Disability Index scores (ODI) were used to evaluate the clinical outcomes. Frankel’s classification system was employed to access the initial and final neurologic function. Fusion of the bone grafting was classified by Bridwell’s grading system.

**Results:**

A total of 55 patients were included in this study with a mean postoperative follow-up time of 2.6 ± 0.8 years (range, 2 to 5). There were 40 males and 15 females with a mean age of 39.8 ± 14.7 years (range, 27 to 57). The Brucella agglutination test was ≥ 1:160 in all patients, but the blood culture was positive in 43 patients (78.1%). A statistical difference was observed in ESR, CRP, VAS, ODI, and JOA between preoperative and final follow-up (*P* < 0.05). Neurological function was significantly improved in 20 patients with preoperative neurological dysfunction after surgery. According to Bridwell’s grading system, the fusion of bone grafting in 48 cases (87.2%) was defined as grade I, and grade II in 7 cases (12.7%). None of the infestation recurrences was observed.

**Conclusion:**

One-stage posterior surgery combined with anti-Brucella therapy was a practical method in the treatment of LBS with severe neurological compression and spinal sagittal imbalance.

## Background

Human brucellosis disease was an infectious zoonotic allergic disease caused by Brucella [1], which was usually transmitted by occupational contact (e.g., veterinarians, slaughterhouses, animal husbandry) and the digestive tract (consumption of contaminated products). It remained a serious public health problem in livestock regions, such as northern China, Australia, the Mediterranean region, and India [2, 3]. A total of 240,000 people worldwide were at risk, with more than 500,000 new cases annually, and 10–85% of patients might be accompanied by involvement of the skeletal system [4–7].

Lumbosacral was the common region of the spinal Brucella spondylitis [8, 9], with an incidence of 2–53% [10], especially L4–5 level, and L5–S1 level [11, 12]. However, the insidious progression of brucellosis lesion made anti-Brucella therapy hardly intervene promptly, resulting in irreversible destruction of the lumbar vertebral body, including abscess formation, disc destruction, and vertebral sclerosis [13]. Failure to diagnose and treat LBS promptly might result in serious sequelae, such as chronic low back pain, neurological dysfunction, and even kyphotic deformity [13, 14]. In clinical practice, hence, the treatment plan for patients with lumbosacral Brucella spondylitis (LBS) combined with spinal cord compression symptoms or kyphotic deformity remains a great challenge for clinicians.

At present, the standard treatment of LBS was non-surgical interventions (antibiotics chemotherapy: doxycycline, rifamycin). Surgical intervention should be considered when the spinal cord compression symptoms or kyphotic deformity occurred, and the principle was to remove the lesion, relieve the spinal cord compression and restore the spinal sagittal balance. When surgery was the treatment of choice, the indication of surgical procedure (anterior, posterior and combined anterior and posterior surgery) remains controversial. Besides, the clinical efficacy of the percutaneous ultrasonic or CT-guided evacuation of paravertebral collections has also been reported [13], but the recurrence of infection still exists since the limited visual field of the surgical procedure. Posterior surgery was suggested since its satisfactory efficacy in removing lesions, decompression, deformity correction, and restoring the spinal sagittal balance, especially for patients with significant lesion destruction and intractable back pain. Therefore, the purpose of this study was to retrospectively analyze the clinical efficacy of patients with LBS managed by one-stage posterior surgery combined with anti-Brucella therapy in our hospital and summarize the surgical indications for the treatment strategy.

## Patients and methods

After receiving written informed consent from participants and approval from the Ethics Committee of our institute, the clinical data of patients with LBS treated by one-stage posterior surgery combined with anti-Brucella therapy were retrospectively collected and evaluated, from June 2010 to June 2020. Inclusion criteria: brucellosis poisoning symptoms [back pain, fever (high “spikes” in the afternoon), night sweats, body-wide aches, headache]; serum agglutination test ≥ 1:160; abscess formation in the paraspinal or psoas muscle; vertebral body disruption, sclerosis of the residual bone and osteophyte formation (“beak” shape of vertebrae anterior edge) confirmed by imaging films; managed by one-stage posterior surgery combined with anti-Brucella therapy; follow-up time > 1 year. Patients were excluded for incomplete medical records, poor compliance, combined with other immune or parasitic diseases, or follow-up time less than 1 year.

The demographic data, pharmacologic treatment records, biopsy or culture results of the cyst, index of C-reactive protein (CRP), and erythrocyte sedimentation rate (ESR) were documented.

### Surgical technique

A posterior midline incision was performed to expose the spinous process, lamina, articular process, and screw insertion entrance point of the diseased vertebra. Two pedicle screws were respectively inserted above and below the lesion after confirming a satisfactory position. Temporary rod fixation was applied to the milder symptom side. Fenestration decompression of the vertebral plate was performed on the side with severe symptoms (part of the superior and inferior facets could be removed if necessary). The intervertebral space was removed thoroughly, and the lesion was sent for pathological examination. Decompression of the vertebral plate fenestration and removal of part of the superior and inferior facets were performed on the compression symptom severer side. For patients with compression symptoms of the double-side nerve root, sneak decompression should be performed on the contralateral side. The base of the spinous process of the vertebral body was removed by the forceps and curette to enlarge the central canal, and the sac should be distracted by a nerve dissector. The cartilage endplate was removed to expose the subchondral bone, and the removed uninfected bone was bitten into small pieces for the mixture with streptomycin. Then these were implanted into the intervertebral space. If the amount of bone graft was insufficient, the autologous iliac bone could be considered for the supplement. Finally, a connecting rod and screw cap were installed, after confirming the satisfactory fixation position by fluoroscopy again. The incision was flushed with sufficient 0.9% saline, a drainage tube was placed in the surgical area, and the incision was closed sequentially.

### Postoperative management

Antibiotics were managed for 2 or 3 postoperative days, and the surgical area drainage tube was removed when drainage volume was < 30 mL/day. Furthermore, the lumbosacral brace was applied for 3 months for helping with postoperative rehabilitation. Anti-Brucella therapy was managed for a minimum of 6 postoperative weeks following the standard WHO-recommended oral regimen: rifampicin (600 mg/day), and doxycycline (200 mg/day). Subsequently, radiography, ESR, and CRP were examined at 1, 6, 12, 18, and 24 postoperative months. All patients were followed up by special recovery questionnaires using the smartphone after being discharged. The visual analogue scale (VAS), Japanese Orthopaedic Association (JOA) and Oswestry Disability Index scores (ODI) were used to evaluate the clinical outcomes. Frankel’s classification system was employed to access the initial and final neurologic function. Fusion of the bone grafting was classified by Bridwell’s grading system.

### Statistical analysis

Data were analyzed by the SPSS 21.0 software package (Chicago, IL, USA). Continuous variables were expressed as mean ± standard deviation (SD), and the distribution of the data was evaluated by the Shapiro–Wilk test. Comparisons between groups (preoperative vs. three postoperative months, and preoperative vs. final follow-up) were performed using the Chi-square test or paired t-test. *P* < 0.05 was considered a statistical significance.

## Results

A total of 55 patients were included in this study with a mean postoperative follow-up time of 2.6 ± 0.8 years (range, 2 to 5). There were 40 males and 15 females with a mean age of 39.8 ± 14.7 years (range, 27 to 57, Table 1). All patients were hampered by lower back pain and limited waist mobility. Further, there were 28 patients (50.9%) with radiating pain in the lower limb and 41 patients (74.5%) with a history of night sweats. Destruction of the vertebral body was observed in 30 patients (54.5%), spinal canal stenosis in 32 patients (58.1%), paravertebral abscess formation in 32 patients (58.1%), paravertebral soft tissue involvement in 27 patients (49%), and epidural granulation tissue or abscess in 19 patients (34.5%). The preoperative serum agglutination test was ≥ 1:160 in all patients and the blood culture was positive in 43 patients (78.1%). Thirty-seven patients (67.2%) were infected with *Brucella melitensis*, 5 patients (9%) with *Brucella abortus*, and one patient (1.8%) with *Brucella suis*. The mean serum levels of ESR and CRP were 41.3 ± 15.5 mm/h (range, 25 to 57), and 33.6 ± 18.5 mg/L (range, 14 to 52) respectively.

**Table 1:** Clinical data of patients

Patient	Age (range, year)	Gender (M/F)	Affected level	Pathogen	Extra-spine infestation	Postoperative grade of FC	Follow-up time (year)	Outcome
1	40–45	M	L2–L3	BM	Fever	E	4	FOD
2	27–32	M	L3–L5	BA	Fever + S	E	3	FOD
3	45–50	F	L4–L5	BM	Fever + H + S	C	3	ND
4	45–50	M	L2–L4	Neg	Fever	E	2	FOD
5	30–35	M	L2–L3	BM	Fever	E	3	FOD
6	32–37	M	L4–L5	BM	Fever	E	5	FOD
7	40–45	M	L2–L3	BM	Fever	E	3	FOD
8	45–50	M	T12–L3	BM	Fever	E	4	FOD
9	35–40	M	L4–L5	BM	Fever	E	2	FOD
10	50–55	F	L5–S1	BA	Fever + H	E	5	FOD
11	40–45	M	L2–L4	BM	Fever	E	3	FOD
12	47–52	M	L3–L4	Neg	Fever	E	2	FOD
13	45–50	F	L3–L5	BM	Fever + H	E	2	FOD
14	35–40	M	T11–L2	BA	Fever + H + S	D	4	FOD
15	40–45	M	L3–L5	BM	Fever	E	2	FOD
16	32–37	M	L4–L5	Neg	Fever + H	E	3	FOD
17	40–45	M	L5–S1	Neg	Fever + H + S	C	4	ND
18	40–45	F	T10–L2	BM	Fever	E	2	FOD
19	40–45	M	L3–L5	BM	Fever + H	E	3	FOD
20	40–45	M	L3	BA	Fever	E	2	FOD
21	30–35	M	S1	BM	Fever	E	5	FOD
22	40–45	F	L5–S1	BM	Fever	E	4	FOD
23	45–50	M	L4–L5	BS	Fever	E	2	FOD
24	42–47	F	L1–L4	BM	Fever	E	2	FOD
25	50–60	M	L3	BA	Fever + H	E	3	FOD
26	42–47	M	L5–S1	BM	Fever	E	2	FOD
27	35–40	M	L3	BM	Fever + H + S	D	2	FOD
28	35–40	M	L5	Neg	Fever + H	E	4	FOD
29	45–50	M	L1–L3	BM	Fever	E	3	FOD
30	32–37	F	T12	BM	Fever	E	2	FOD
31	40–45	M	L2–L3	Neg	Fever	E	3	FOD
32	35–40	F	T12	BM	Fever	E	2	FOD
33	25–30	M	L1–L2	BM	Fever + H	E	4	FOD
34	38–42	M	L2–L4	BM	Fever	E	2	FOD
35	35–40	M	T12–L2	BM	Fever + H + S	E	3	FOD
36	38–42	F	L1–L3	Neg	Fever	E	2	FOD
37	35–40	M	L4–L5	BM	Fever	E	4	FOD
38	25–30	F	L5–S1	BM	Fever + H	E	3	FOD
39	25–30	F	T12–L2	Neg	Fever	E	5	FOD
40	30–35	M	L3–L5	BM	Fever	E	2	FOD
41	45–50	M	T12	BM	Fever + H + S	E	3	FOD
42	42–47	M	S1–S2	BM	Fever + H	E	3	FOD
43	52–57	M	L5–S1	BM	Fever	E	4	FOD
44	42–47	M	L2–L3	Neg	Fever	E	3	FOD
45	35–40	F	T12–L2	BM	Fever	E	3	FOD
46	32–37	M	L4–L5	BM	Fever + H	E	2	FOD
47	45–50	F	L4–L5	BM	Fever + H + S	E	3	FOD
48	30–35	F	T12–L2	BM	Fever + H + S	E	4	FOD
49	40–45	M	L2–L3	Neg	Fever + H + S	E	3	FOD
50	40–45	M	L3–L5	BM	Fever + H	E	2	FOD
51	45–50	M	L5–S1	Neg	Fever	E	4	FOD
52	45–50	F	T12–L2	BM	Fever	E	3	FOD
53	40–45	M	L2–L3	Neg	Fever	E	3	FOD
54	30–35	M	L2–L4	BM	Fever	E	2	FOD
55	28–32	M	L5	BM	Fever + H	E	5	FOD

BA, *Brucella abortus*; BM, *Brucella melitensis*; BS, *Brucella suis*; F, female; FOD, free of disease; H, hepatomegaly; M, male; Neg, negative; ND, neurological dysfunction; S, splenomegaly

**Fig. 1:**
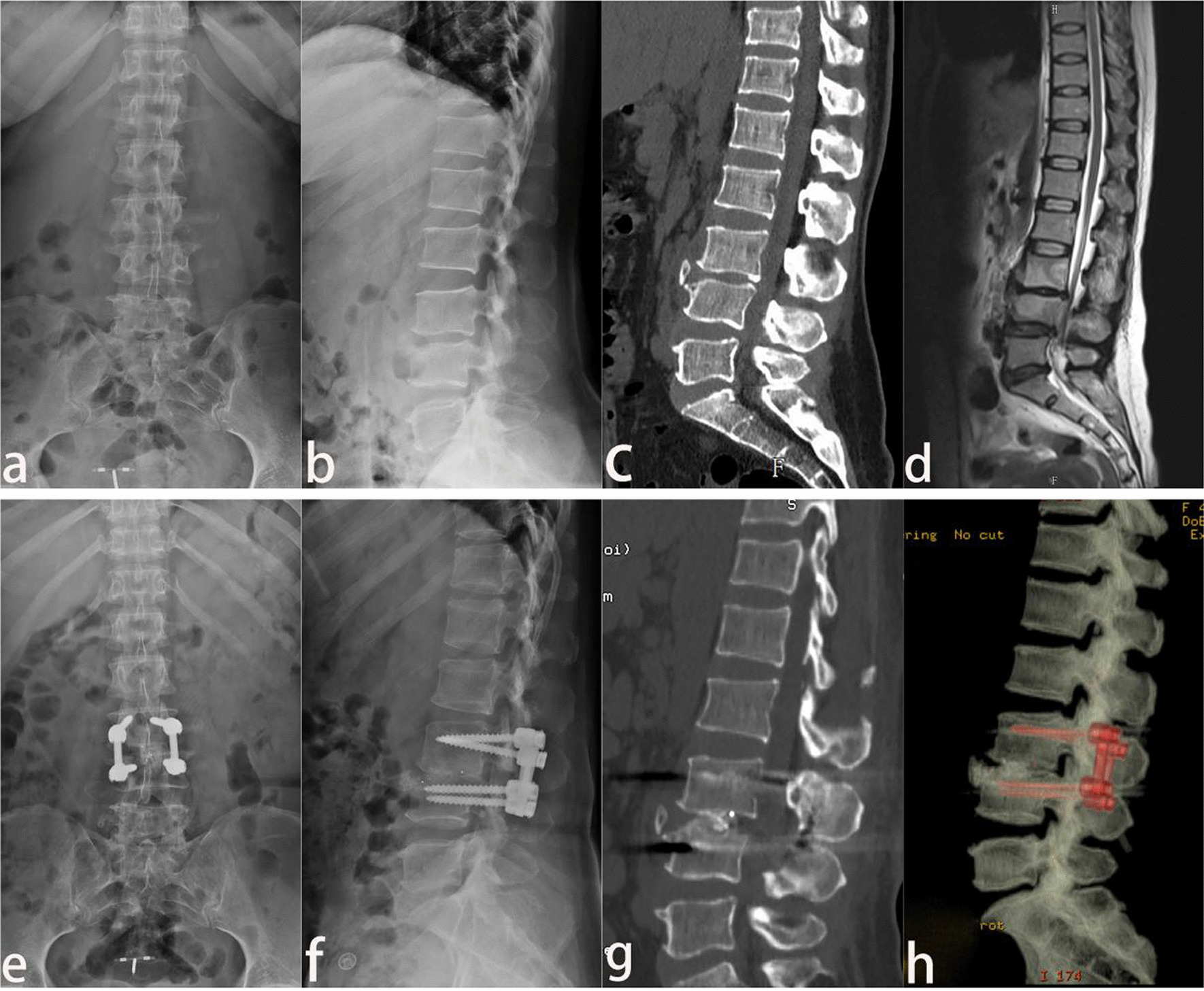
A 44-year-old female with lumbosacral Brucella spondylitis. **a**–**d** The lesion of the lumbosacral spine (L3, L4) was shown by the preoperative positive and lateral X-ray, CT sagittal reconstruction, and MRI. **e**, **f** The vertebral body was fixed firmly by the screw at 3 postoperative months, which was presented by X-ray. **g**, **h** CT sagittal and three-dimensional reconstruction demonstrated that the lesion was removed completely, and the internal fixation was stable without recurrence of the lesion at 6 postoperative months

**Fig. 2:**
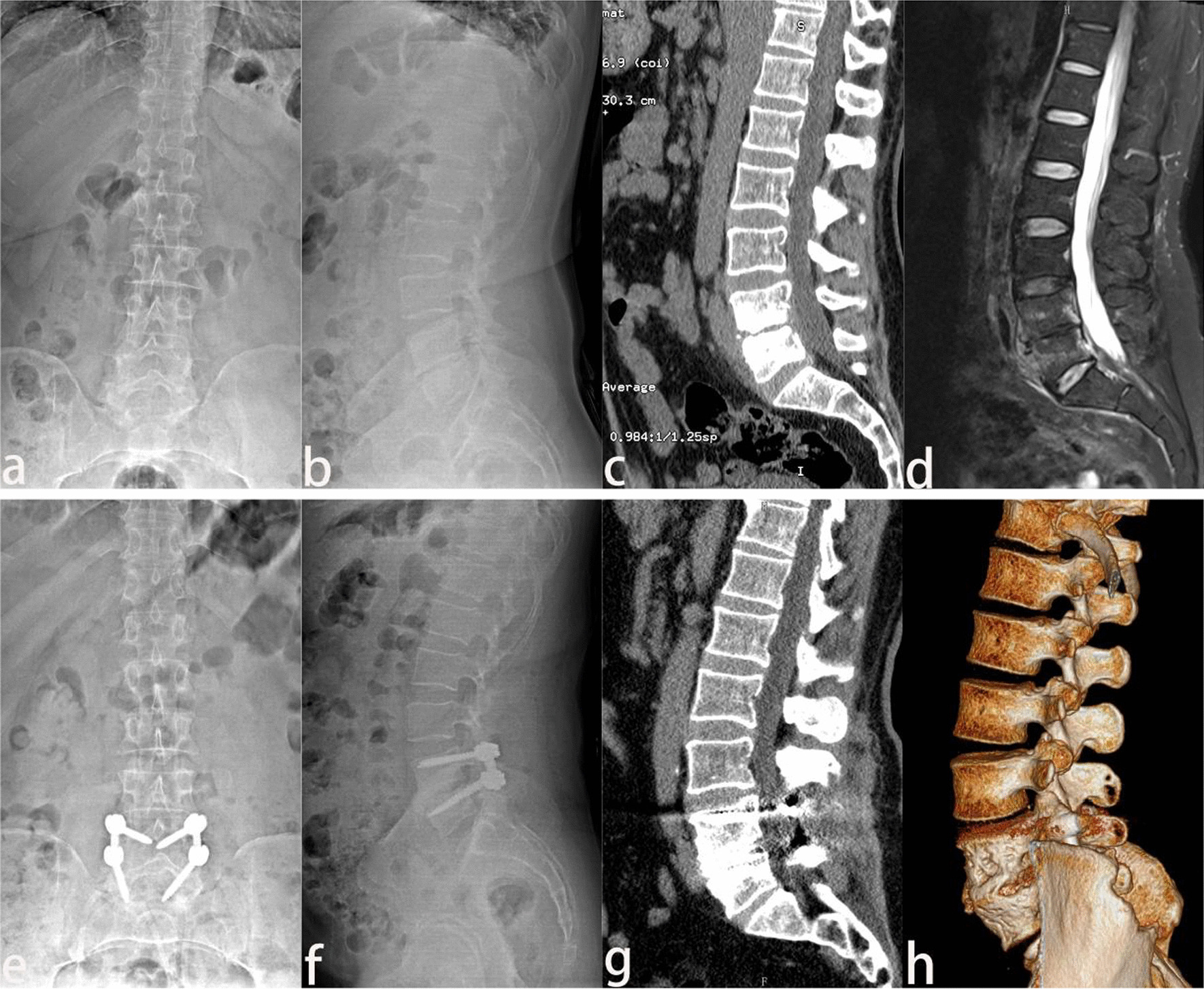
A 57-year-old female with lumbosacral Brucella spondylitis. **a**–**d** L5, S1 vertebral body destruction, and intervertebral space narrowing caused by infection were indicated by the anteroposterior and lateral X-ray, CT sagittal reconstruction, and MRI. **e**, **f** The vertebral body was fixed firmly by the screw at 3 postoperative months, which was presented by X-ray. **g**, **h** CT sagittal and three-dimensional reconstruction demonstrated that the lesion was removed completely, and internal fixation was in a satisfactory position without recurrence of infection at the 6 postoperative months

The poisoning symptoms were relieved in all patients after posterior surgery combined with anti-Brucella therapy, without local spine tenderness or percussion pain at follow-up. The mean operation time was 138.7 ± 63.8 min (range, 75 to 205) with a mean intraoperative blood loss of 215.4 ± 77.1 mL (range, 135 to 300). The average hospitalization time was 12.7 ± 6.2 days (range, 6 to 19). ESR, CRP, VAS, ODI, and JOA were improved after surgery, and a statistical difference was observed between preoperative and final follow-up (*P* < 0.05, Table 2). The typical cases described in this study were referred to in Figs. 1 and 2.

**Table 2:** Comparison of preoperative, postoperative VAS, ODI, JOA scores, and inflammatory indicators

Variable	Preoperative	Three postoperative months	Final follow-up	Improvement rate (%)
ESR	41.35 ± 15.50	9.15 ± 3.17*	7.31 ± 2.34*^#^	91.6
CRP	33.61 ± 18.54	5.18 ± 1.79*	2.04 ± 0.71*^#^	86.3
VAS	6.04 ± 1.49	1.69 ± 0.57*	0.72 ± 0.53*^#^	92.8
ODI(%)	54.08 ± 9.92	15.87 ± 5.93*	10.44 ± 5.04*^#^	83.1
JOA	15.12 ± 3.89	23.47 ± 3.13*	25.43 ± 3.49*^#^	80.5

*CRP* C-reactive protein, *ESR* Erythrocyte sedimentation rate, *JOA* Japanese Orthopaedic Association, *ODI* Oswestry disability index, *VAS* Visual analogue scale

*Comparison of preoperative, *P* < 0.05

^#^Comparison of three postoperative months, *P* < 0.05

Neurological function was significantly improved in 20 patients with preoperative neurological dysfunction after surgery. In short, two patients with preoperative Frankel’s grade C recovered to grade D at 1 postoperative month, and one patient with preoperative Frankel’s grade C recovered to grade E at 6 postoperative months. Seven of the 17 patients with Frankel’s grade D recovered to grade E at 1 postoperative month, and the remaining cases recovered gradually to grade E at the follow-up. Only 2 patients with preoperative neurological dysfunction (Frankel’s grade C) were not improved after surgery (Table 3). The mean fusion time was 6.9 ± 0.7 months (range, 6 to 8). According to Bridwell’s grading system, the fusion of bone grafting in 48 cases (87.2%) was defined as grade I, and grade II in 7 cases (12.7%). None of the internal fixation loosening and breakage was found during the follow-up.

**Table 3:** Comparison of neurological outcomes after surgery

Frankel’ grade*	Preoperative	One postoperative month	Three postoperative months	Six postoperative months	Final follow-up
A	0	0	0	0	0
B	0	0	0	0	0
C	3	1	0	0	0
D	17	12	5	3	2
E	35	42	50	52	53

*Frankel classification

## Discussion

The pathological basis of LBS was chronic degeneration of the intervertebral disc and vertebral bone destruction, and intractable back pain as the main clinical manifestations [15]. Intervertebral space stenosis was the common presentation of radiography, presented in 32 patients (58.1%) in this study. In the view of anatomy, the intervertebral joint was the stress concentration area behind the spine, which might be easily affected by intervertebral space stenosis. The lesions might slowly invade the articular surface of the vertebrae body and resulted in the proliferation and hardening of the articular surface when the anti-Brucella therapy was not intervened timely. The biomechanical structure stability of the intervertebral joint and spine sagittal balance might be destroyed if the progression continued. Via published studies [16–18], the phenomenon that invasion of the synovium and cartilage surface of joints by Brucella was more common. Posterior joint destruction combined with disc degeneration might result in vertebral slippage. On that occasion, intractable back pain could be worsened by spinal sagittal imbalance and severe vertebral slippage, as well as the injury of the nerve root. Fortunately, the velocity of infiltrative bone destruction in Brucella infestation was slow. The process of bone destruction was accompanied by the process of bone repair, so the sequestrum was not commonly formed [17, 19]. Hence, the preservation of the vertebrae’s structural morphology was a special character of LBS, which was different from spinal tuberculosis [19]. The spinal stability of patients with LBS was usually better than that of spinal tuberculosis, and that’s why kyphotic deformity was rare among patients with LBS.

Blood culture remained the gold standard for diagnosis of Brucella infestation [1, 3]. Yet, the sensitivity of blood culture depended on several factors, especially the disease phase and previous antibiotics usage. In the acute phase, the sensitivity of blood culture might be more than 80%, while for patients with chronic infestation, its sensitivity was approximately 30–70% [20]. Although the population was susceptible to Brucella, most of the clinical symptoms could be effectively relieved by prompt and standard antibacterial therapy [21, 22]. The indications and timing of surgical intervention were still controversial. But the current recognition was that the surgery should be prepared for patients whose Brucella poisoning symptoms cannot be effectively improved by anti-Brucella therapy (6 weeks of medication and 1 week of drug withdrawal), and combined with one of the following symptoms [7, 23, 24]: paravertebral abscess or psoas abscess; intervertebral disc destruction; spinal structure instability; accompanied by other bacterial infections.

The purposes of surgery were radically removing the lesion, improving the local blood circulation, relieving the nerve root compression symptoms, and restoring the spinal sagittal balance to promote the early limbs’ function recovery. At present, the surgical procedures mainly consisted of anterior, posterior, and combined anterior and posterior surgery. The choice of approach should be based on the location of the spinal lesion, degree of vertebral destruction, level of spinal nerve compression, and surgeon’s technical proficiency. It had been reported in the literature that posterior surgery was more practical for intraspinal granulation and abscess removal, especially for patients with intraspinal nerve damage caused by posterior column lesions. While the combined anterior and posterior surgery was recommended for patients with perivertebral abscess, psoas abscess, or greater anterior column destruction [7]. In this study, the posterior surgery was successfully performed in all patients, since the paravertebral abscess combined with spinal nerve compression symptoms occurred in most patients (83.6%). According to Frankel’s classification, the spinal nerve compression symptoms were improved from grade C/D to grade E in 53 patients (96.3%) at the final follow-up.

To our knowledge, anterior surgery had also been recommended by previous studies. However, this method not only required meticulous surgical technique with a prolonged operative time but also left the risk of damaging the iliac vessels and sympathetic nerves of the complex anatomy of the anterior lumbosacral spine. Yin et al. [25] reported a case series of 16 patients with Bucella spondylitis managed by anterior surgery with a mean operation time and intraoperative blood loss of 237.4 min and 580.2 mL, respectively. In this cohort, the mean operation time was 138.7 min (range, 80 to 200) with a mean intraoperative blood loss of 215.4 mL (range, 60 to 370), which was significantly less than Yin’s study. Additionally, there was no back pain caused by iatrogenic, and the fusion of bone grafting in 48 cases (87.2%) was defined as grade I, and grade II in 7 cases (12.7%).

Although the posterior surgery made up for the lack of anterior surgery, the spinal sagittal imbalance caused by serious destruction of the anterior column could not be ignored. The persistent nerve compression symptoms (back pain or numbness) might also be caused by the long period of the insidious development of vertebrae destruction. The intervertebral space and the upper and lower endplates of adjacent vertebral bodies were usually involved, but the distribution of abscesses was limited, which rarely exceeded the edge of the vertebral body [1, 6]. A retrospective comparative study published by Ulu-Kilic et al. [4] also showed that the extent of paravertebral abscesses in thoracolumbar Brucella spondylitis generally did not exceed the upper and lower edges of the destroyed vertebral body. Some patients with nerve root compression symptoms caused by intervertebral discs bulging from intraspinal abscesses or swelling could also be effectively treated by prolonging the antibacterial therapy period. Thus, the completeness of lesion removal should not be overemphasized [23]. Chen et al. [8] reported that 24 patients with Brucella spondylitis were treated with one-stage posterior surgery and received satisfactory postoperative results. In this study, the neurological compression symptoms of two patients were not improved. We considered that the irreversible nerve damage might be caused by their long period of chronic infestation. In our experience, hence, the earlier anti-Brucella therapy intervention, the less incidence of vertebrae destruction and neurological compression symptoms. Clinicians in the endemic area should become aware of brucellosis in the differential diagnosis of febrile diseases with peculiar musculoskeletal to prevent the increased medical burden. Yet, it was necessary to perform the surgery when the spinal sagittal imbalance occurred caused by the development of infestation.

ESR and CRP returned to a normal level in the 3rd postoperative month (*P* < 0.05). In the comparison of the preoperative, the pain symptoms and neurological dysfunction were improved (*P* < 0.05). In our opinion, posterior surgery was recommended for patients without neurological dysfunction to effectively avoid excessive damage to the structure of the posterior column of the spine, which also decreased the risk of intraoperative injury to the nerve roots and dissemination of infection. Besides, the surgical procedure was suggested to be performed on the severer side for hemi-spinal fenestration and resection of the facet joint selected for patients with neurological compression symptoms. Once the severe side was completely decompressed, it was easy to decompress the mild side. The decompression should be carefully manipulated to avoid the fracture of the contralateral lamina or excessive destruction of the facet joints.

Last but not the least, the results of this study might be affected by potential limitations since its retrospective and single-centre nature. There was also no standardized surgical method for the treatment of advanced LBS. Hence, a prospective study with larger-sample and multi-centre would be helpful for the management of LBS.

## Conclusion

Standard anti-Brucella therapy was indispensable for infestation control in the early stage of LBS. One-stage posterior surgery combined with anti-Brucella therapy was a practical method in the treatment of LBS with severe neurological compression and spinal sagittal imbalance.

## Data Availability

The data sets generated and analyzed during the current study are not publicly available due to restrictions on ethical approvals involving patient data and anonymity but can be obtained from the corresponding author at reasonable request.

## Abbreviations

CRP: C-reactive protein
ESR: Erythrocyte sedimentation rate
JOA: Japanese Orthopaedic Association
LBS: lumbosacral brucellosis spondylitis
ODI: Oswestry Disability Index
VAS: Visual Analogue Scale
